# Adjunctive Antiseptic Irrigation of Periodontal Pockets: Effects on Microbial and Cytokine Profiles

**DOI:** 10.3390/dj8040124

**Published:** 2020-11-02

**Authors:** Anton Vitt, Andrei Babenka, Elisabeth A. Boström, Anders Gustafsson, Ronaldo Lira Junior, Veronica Slizen, Timo Sorsa, Taina Tervahartiala, Kåre Buhlin

**Affiliations:** 1 Department of Dental Medicine, Division of Periodontology, Karolinska Institutet, 14152 Huddinge, Sweden; elisabeth.bostrom@ki.se (E.A.B.); anders.gustafsson@ki.se (A.G.); ronaldo.lira.junior@ki.se (R.L.J.); timo.sorsa@helsinki.fi (T.S.); kare.buhlin@ki.se (K.B.); 2 First Department of Therapeutic Dentistry, Belarusian State Medical University, 220045 Minsk, Belarus; 3 Department of Bioorganic Chemistry, Belarusian State Medical University, 220045 Minsk, Belarus; labmdbt@gmail.com; 4 Department of Microbiology, Virology and Immunology, Belarusian State Medical University, 220045 Minsk, Belarus; veronal@tut.by; 5 Department of Oral and Maxillofacial Diseases, Institute of Dentistry, University of Helsinki, 00290 Helsinki, Finland; taina.tervahartiala@helsinki.fi

**Keywords:** inflammation, periodontitis, chlorhexidine, polyhexamethylene guanidine phosphate, irrigation

## Abstract

To evaluate the effect of adjunctive antiseptic irrigation of periodontal pockets on microbial and cytokine profiles. Fifty-nine patients with severe periodontitis were allocated to one of three groups for scaling and root planing facilitated with different adjunctive antiseptics: 1% polyhexamethyleneguanidine phosphate (PHMG-P) (n = 19), 0.2% chlorhexidine (CHX) (n = 21) or distilled water (n = 19). Gingival crevicular fluid and subgingival bacterial samples were collected at baseline, and at 2 weeks, and 1 and 4 months. The levels of interleukin (IL)-1β, IL-8, IL-10, and IL-17A, matrix metalloproteinase (MMP)-8, *Porphyromonas gingivalis*, *Tannerella forsythia*, *Treponema denticola*, *Fusobacterium nucleatum,* *Aggregatibacter actinomycetemcomitans*, and *Prevotella intermedia* were determined. There were no intergroup differences in cytokine concentrations and bacterial counts at any follow-up, however, varying patterns were observed. In the PHMG-P and water groups IL-1β expression peaked at 2 weeks and then gradually declined. In all three groups, the dynamics of MMP-8 concentration were non-linear, increasing by 2 weeks and then declining to below baseline (*p* > 0.05). *P. gingivalis* and *T. forsythia* declined within the first month and increased thereafter, not regaining the baseline level. Adjunctive antiseptic treatment was associated with changes in biomarkers and bacterial counts in the course of the study. The effects of adjunctive antiseptic irrigation were limited in the applied protocol.

## 1. Introduction

The permanent presence of microbial flora in the human oral cavity is beneficial and is essential to health [1]. However, bacteria may also act as causal agents of oral diseases [2]. Dental biofilm is recognized as a cause and significant risk factor for periodontal diseases [3]. The role of *Aggregatibacter actinomycetemcomitans*, *Porphyromonas gingivalis*, and *Tannerella forsythia* is generally accepted and they are regarded as ‘consensus’ periodontopathogens. *Eubacterium nodatum*, *Fusobacterium nucleatum*, *Prevotella intermedia*, *Prevotella nigrescens*, and *Treponema denticola* are strongly associated with chronic periodontitis [4].

After binding to the pathogen-sensing system, components of the bacterial cell, including lipopolysaccharide (LPS), through intracellular signaling cascades, activate the release of large numbers of pro-inflammatory cytokines, including tumor necrosis factor (TNF)-α and interleukin (IL)-1β, which in turn recruit leukocytes to the sites of periodontitis, in order to combat infection [5,6]. Subsequently leukocytes degranulate substantial amounts of inflammatory cytokines such as TNF-α, IL-1β, and IL-6. Production of the receptor activator of nuclear factor-kB ligand (RANKL) disrupts the balance in the level of osteoclast differentiation factor, causing osteoclastogenesis, ultimately leading to increased resorption of alveolar bone [7]. The local inflammatory reaction also affects production and release of matrix metalloproteinases (MMPs). MMPs are genetically distinct but structurally related proteolytic, zinc-dependent endopeptidases, belonging to the collagenase family [8]. MMPs can be expressed by neutrophils, fibroblasts, epithelial cells, macrophages, etc. [9,10,11,12]. In vitro, IL-1β induces MMP-8 expression by gingival fibroblasts [10]. Neutrophil degranulation and MMP-8 release are regulated by cytokines (IL-1β, TNFα) and depend on bacterial stimulus [8]. In contrast to the destructive pathway involving primarily pro-inflammatory cytokines, regulatory pathways mediated by anti-inflammatory cytokines IL-10 and IL-4 can control or attenuate the progression of periodontitis [13,14,15].

As microbial plaque is strongly implicated in periodontal disease, antimicrobial drugs may help reduce the number of periodontopathogenic and associative microbial species [15].

Polyhexamethylene guanidine (PHMG) derivatives referred to the guanidine polymers have been widely applied for many years as antiseptics in medicine and industry [16]. Polyhexamethylene biguanide (PHMB) interacts with phospholipids in the bacterial membrane, causing increased fluidity, permeability, and loss of integrity, followed by the death of the organism [15,17,18]. Structurally similar conjugates, such as polyhexamethylene biguanide hydrochloride (PHMB-H) and polyhexamethylene guanidine phosphate (PHMG-P), are synthesized by incorporating different anions into the PHMG molecule. PHMB-H has been extensively tested in vivo and in vitro. In the form of a mouthwash, PHMB-H consistently inhibited plaque regrowth and reduced oral bacterial counts, indicating its potential application as an active ingredient in dentifrices [19,20,21].

Polyhexamethylene guanidine phosphate (PHMG-P) has been recently proposed for control of infections in dentistry. To date, there are few reports dealing with the impact of PHMG-P on biomarkers and microbial profiles in periodontal pockets.

The aim of this study is to compare the effects of adjunctive PHMG-P and CHX irrigation on the microbial and cytokine profiles of periodontal pockets.

## 2. Materials and Methods

The study is based on samples obtained from volunteers who participated in the clinical trial [22]. Briefly, 59 patients (30 males and 29 females) with severe periodontitis, possessing at least 3 teeth with periodontal pockets ≥ 6 mm deep, and radiographic evidence of extensive bone loss (≥one third of root length) were enrolled in the study [15]. According to a new classification of periodontal and peri-implant diseases, such condition is referred to as chronic periodontitis stage III or IV grade B [23]. The Armitage 1999 diagnostic terms were used because they were widely distributed at the time of the study [24]. All tested subjects were randomly allocated to one of three treatment groups for scaling and root planing (SRP), using different adjunctive irrigants: Aquin (Inkraslav©, Minsk, Belarus), containing PHMG-P 1% as the active substance (19 subjects), 0.2% chlorhexidine (Public Pharmaceutical Service, Minsk, Belarus) (21 subjects) and distilled water (19 subjects). After the baseline examination, all patients received initial periodontal therapy, which included motivation, oral hygiene instruction and full-mouth debridement, using a combination of ultrasonic and manual instrumentation, and irrigation with Aquin, 0.2% chlorhexidine or distilled water. At each follow-up appointment, all treatment procedures were repeated and every periodontal pocket was irrigated. After baseline, five following appointments were scheduled: upon 2 weeks, and 1, 4, 6, and 12 months. GCF and bacterial samples were collected at baseline, 2 weeks, 1 month, and 4 months after the initial intervention.

The study was conducted according to the principles outlined in the Declaration of Helsinki on experimentation involving human subjects and approved by the resolution N.5 of the Ethical Board of Belarusian State Medical University on 18 April, 2011. Prior taking part in this research every patient was provided with written and oral information about the study, the substances to be used, and treatment methods. Written informed consent was granted by every person recruited in the study. Participation in the study was voluntary and the patients could withdraw at any time. All personal data were encoded, and the keys were kept separately from the codes. As a part of the treatment routine, all patients were informed about examination results and diagnosis [15].

### 2.1. Gingival Crevicular Fluid (GCF) Sampling

In order to avoid bleeding, GCF samples were collected before bacterial sampling. Each tooth was isolated with cotton rolls and air dried. The contents of the periodontal pockets were extracted by inserting paper strips PerioPapers^®^ (Oraflow Inc., New York, NY, USA) for 30 s into four periodontal pockets, representing each quadrant. The same periodontal pockets were used for sampling at each follow-up. Strips contaminated with blood were discarded. The strips with samples were then placed individually into test tubes, frozen rapidly at −20 °C, transferred to a freezer within a week and maintained at −80 °C until further processing.

### 2.2. Collecting of Subgingival Biofilm Samples

Bacteria were sampled by scaling from the deepest four periodontal pockets, each representing a quadrant of the dentition, pooled with sterile transport medium in test tubes, then frozen at −20 °C to await DNA extraction.

### 2.3. Biomarker Immunoassays

The levels of IL-1β, IL-8, IL-10, and IL-17A were determined by multiplex immunoassay, according to the manufacturer’s instructions (ProcartaPlex^TM^ Multiplex Immunoassay, ThermoFisher, Vienna, Austria). The readings were recorded using Bio-Plex 100 (Bio-Rad Laboratories, Inc., Hercules, CA, USA). The assay range was as follows IL-1β: 0.32–1330 pg/mL; IL-10: 0.18–750 pg/mL; IL-17A: 0.25–1030 pg/mL; IL-8: 2.25–9200 pg/mL.

MMP-8 levels were measured by time-resolved immunofluorometric assay (IFMA) as described earlier [10,25]. Briefly, the MMP-8 specific monoclonal antibodies 8708 and 8706 (Medix Biochemica, Kauniainen, Finland) were applied as a capture antibody and a tracer antibody. The tracer antibody was labeled with europium-chelate. The assay buffer contained 20 mM Tris-HCl (pH 7.5), 0.5 M NaCl, 5 mM CaCl_2_, 50 μM nCl_2_, 0.5% bovine serum albumin, 0.05% sodium azide, and 20 mg/l diethylenetriaminepentaacetic acid. GCF samples were diluted in assay buffer and incubated for 1 h, followed by another 1 h incubation with tracer antibody. Enhancement solution was added and after 5 min incubation fluorescence was measured using Delfia Research Fluorometer (Wallac, Turku, Finland). The detection limit for this assay was 0.08 ng/mL.

Due to the heterogeneity of the samples i.e., variation due to manual collection, GCF samples were normalized to the total protein concentration, measured in each sample using a fluorescence-based assay, according to the manufacturer’s instructions (Qubit Fluorometer, Life Technologies, Carlsbad, CA, USA). Immune marker concentrations were adjusted to total protein concentration and expressed as the ratio (pg of immune marker/mg of total protein).

### 2.4. DNA Extraction and Quality Control

DNA was extracted from clinical samples using “Nucleosorb-C” kit (Primetech, Minsk, Belarus) according to the manufacturer’s instructions. DNA quality and concentration were estimated by spectrophotometry at wavelengths 260/280 nm, 260/230 nm, 260/320 nm (SOLAR PB2201, Minsk, Belarus). The majority of samples reached values 1.7–1.9 at λ = 260/280 nm and molar concentrations >5 ng/µL. Some DNA samples were analyzed with 1% agarose gel electrophoresis to evaluate the degree of fragmentation (mild condition to avoid denaturing) [15].

### 2.5. Primers and Probes for Real-Time PCR

Oligonucleotide sets, including probes developed and validated by other researchers, were used in this study (Table 1) [26,27,28,29]. To optimize the reaction and increase its efficiency, we reserved the right to adjust some sequences (Table 1). They were partially modified with locked nucleic acid (LNA) nucleotides. In each case, we selected the ratio of oligonucleotides in the reaction mixture, and also optimized the buffer conditions to solve secondary structures revealed by “The mfold Web Server” and “Oligoanalyzer” (Integrated DNA Technologies, Inc., Corralville, IA, USA) [30]. The oligonucleotide sequences and modifications are shown in Table 1.

### 2.6. Real Time PCR

Real time PCR was conducted under control of CFX96touch (Bio-Rad, Hercules, CA, USA) according to the following protocol. The reaction mix (25 µL volume) contained 500 nM of each oligonucleotide, including the probe, 2 mM MgCl_2_, 0.1 mM each of dNTP, 1x PCR commercial buffer without MgCl_2_, 1.25 U of thermostable Taq DNA-polymerase without hot start, deionized RNAse/DNAse free water for PCR. The cycling conditions were +95 °C for 3 min—primary denaturation, then 50 cycles: +95 °C for 10 s and +60 °C for 59 s (annealing and elongation steps).

### 2.7. Standard Curves and Reaction Efficiency

To construct standard curves for microbes the mixture of 10 different DNA samples was used with the highest concentrations resulting from DNA purification [15]. Before DNA purification, each sample was collected from a separate patient. This approach is intended to minimize the effect of the sample type and the isolation method on the reaction efficiency.

Quantitation cycle (Cq) values were determined in DNA samples and those with Cq < 20 were selected. The DNA was then pooled and mixed in one tube, vortexed and serially diluted 5-fold, yielding five calibrators with 5-fold steps of DNA concentration. The data on reaction efficiency and R^2^ parameters are presented in Table 2. In all cases the recorded R^2^ values exceeded 0.99, which is consistent with the data reported in the literature. At the same time, in most of the experiments the recorded efficiency of the reactions was superior.

### 2.8. PCR Data Analysis

Real time PCR data were analyzed using BioRad CFX96-touch basic software. The Cq data were converted to MS Excel format for subsequent analysis.

### 2.9. Statistical Analyses

Descriptive statistical data were computed and expressed as the mean ± standard deviation (SD). The Friedman test was used to reveal differences within treatment groups and the Kruskal–Wallis test served for intergroup comparisons along with post-hoc Dunn–Bonferroni test to find differences between groups. To estimate the significance of intergroup differences, the Mann–Whitney test was applied to independent groups and the Wilcoxon signed rank test to dependent groups. Spearman’s rank correlation coefficient was chosen to evaluate the interrelationship of the observed parameters. *p*-value = 0.05 was defined as statistically significant. Data processing was undertaken using the software package SPSS Statistics 25 (IBM©, SPSS© Statistics, Armonk, NY, USA).

## 3. Results

**Figure 1:**
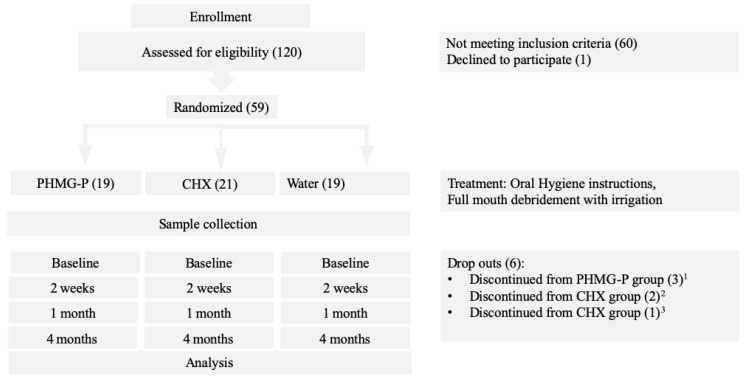
Study flow chart.

Fifty-three patients completed the study while six withdrew, resulting in an overall retention rate of 89.8%. A flowchart of the study design is presented in Figure 1.

The studied inflammatory biomarkers (IL-1β, IL-8, IL-10, IL-17A, MMP-8) and bacterial counts (*P. gingivalis*, *T. denticola*, *F. nucleatum*, *T. forsythia*, *A. actinomycetemcomitans*, and *P. intermedia*) did not show statistical differences between the treatment groups (PHMG-P, CHX, and water) at any follow up. However, varying alteration patterns were recorded (*p* > 0.05).

### 3.1. Dynamics of Biomarkers

**Figure 2:**
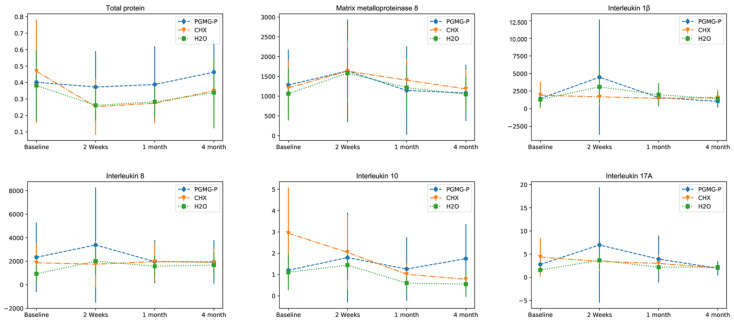
Dynamics of biomarkers in the course of the study.

Total protein concentration followed a similar pattern in all the groups, decreasing from the baseline value to the 2-week time-point and then gradually increasing up to 4 months (Figure 2). The concentration of total protein in the PHMG-P group was rising from the baseline level, whereas in the CHX and water groups it remained below the initial titer. Within the CHX group, there was a considerable reduction in the amount of total protein between baseline and 2-week follow-up examinations (*p* ≤ 0.05), while no significant changes were observed in the PHMG-P and water groups during the study (*p* > 0.05).

The levels of interleukin (IL)-1β, IL-8, IL-10, IL-17A, and matrix metalloproteinase (MMP)-8 were evaluated in 59 patients with severe periodontitis, allocated to one of three treatment groups for scaling and root planing with adjunctive irrigants: Aquin (1% polyhexamethyleneguanidine phosphate (PHMG-P)) (n = 19), 0.2% chlorhexidine (CHX) (n = 21), or dH_2_O (n = 19). Cytokines were assessed using multiplex immunoassay. MMP-8 was investigated by time-resolved immunofluorometric assay. The Friedman test was used to reveal differences within treatment groups. Kruskal–Wallis Test served for evaluation of intergroup differences. To estimate the significance of pairwise intergroup differences, the Mann–Whitney test was applied to independent groups and the Wilcoxon signed rank test to dependent groups. *p*-value = 0.05 was defined as statistically significant.

IL-1β production in the PHMG-P group significantly decreased between 2-week and four-month follow-ups (*p* ≤ 0.05).

IL-1β production significantly increased in water group between baseline and 2-week time points.

IL-17A production in the PHMG-P group significantly decreased between 2-week and 4-month follow-ups (*p* ≤ 0.05).

The concentration kinetics of MMP-8 were nonlinear in all three groups during the study (Figure 2). At the 2-week follow-up MMP-8 increased then gradually declined to below the baseline level, but the changes were not significant (*p* > 0.05).

IL-1β production in the PHMG-P and the water groups peaked at the 2-week time-point and declined under the initial level in the case of the PHMG-P group, and slightly above it in the control group. Pairwise comparison revealed evident changes in PHMG-P group between two-week and four-month examinations and in water group between baseline and two-week follow-ups (*p* ≤ 0.05) (Figure 2). In the CHX group, the cytokine level slid down steadily until the end of the first month and then increased slightly (*p* > 0.05).

IL-8 is a chemokine, a biochemical attractant for neutrophils [32]. The dynamics of IL-8 concentration were in accordance with IL-1β levels in the corresponding groups (Figure 2). The chemokine showed elevated values at 2-week time-point in the PHMG-P and water groups, and then gradually declined by the 4-month time-point. IL-8 production displayed negligible fluctuations in the CHX group, remaining relatively constant. No intragroup differences in IL-8 level were detected at any examination (*p* > 0.05).

IL-10 is regarded as an anti-inflammatory cytokine and produced by many immune cells, e.g., macrophages and regulatory T-cells [33]. It affects dendritic cells, macrophages, and several T-cell subsets. As an anti-inflammatory cytokine IL-10 regulates the cellular immune response. It seems that production of IL-10 was synchronized with the dynamics of the pro-inflammatory cytokines (IL-1β, IL-8, and IL-17A) to balance the strength and type of immune response (Figure 2). No difference within the groups was observed in IL-10 concentration dynamics in the course of the study (*p* > 0.05). Initially the IL-10 level in the CHX group was about 3 times higher than in the other groups but it tended to decline with time. In the PHMG-P and water groups, IL-10 rose from baseline to week 2, then gradually fell until the 4-month in the water group.

IL-17A is produced by the T helper 17 subset of T cell population [34]. The receptor binding IL-17A controls local tissue inflammation and up-regulates a wide range of pro-inflammatory cytokines and chemokines [35]. The level of IL-17A decreased steadily in the CHX group and varied considerably in the PHMG-P group and insignificantly in the water group (Figure 2).

### 3.2. Dynamics of Periopathogens

**Figure 3:**
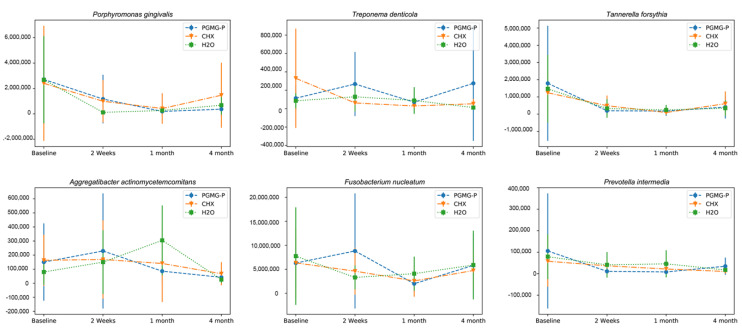
Dynamics of periopathogenic bacteria in the course of the study.

On average, *P. gingivalis* was detected in 70.2% of patients suffering from severe periodontitis. The concentration changed in a similar way in all treatment groups during the study (Figure 3). It was sequentially decreasing in the PHMG-P and CHX groups till the expiry of first month. In the water group the *P. gingivalis* titer fell more sharply within the first fortnight. In the 1- to 4-month span, the bacterial count resumed an upward trend, but failed to rise up to the baseline level. The changes were significant within the PHMG-P group between baseline and 1-month follow-up examinations (*p* ≤ 0.05).

The quantity of *Porphyromonas gingivalis*, *Tannerella forsythia*, *Treponema denticola*, *Fusobacterium nucleatum*, *Aggregatibacter actinomycetemcomitans*, and *Prevotella intermedia* were evaluated by real-time polymerase chain reaction in 59 patients with severe periodontitis, allocated to one of three groups for scaling and root planing with adjunctive irrigants: Aquin (1% polyhexamethyleneguanidine phosphate (PHMG-P)) (n = 19), 0.2% chlorhexidine (CHX) (n = 21), or dH_2_O (n = 19). The Friedman test was used to reveal differences within treatment groups and the Kruskal–Wallis test served for intergroup comparisons. To estimate the significance of intergroup differences, the Mann–Whitney test was applied to independent groups and the Wilcoxon signed rank test to dependent groups. *p*-value = 0.05 was defined as statistically significant.

The concentration of *P. gingivalis* significantly decreased within the PHMG-P group between baseline and 1-month follow-up examinations (*p* ≤ 0.05).

The changes of *T. forsythia* were significant in the group treated with CHX (*p* ≤ 0.05).

At baseline, *T. denticola* was detected on average in 68% of the patients. Mean microbial concentration in the CHX group gradually decreased by 2 weeks and 1 month, followed by a slight increase, but did not regain the baseline level (Figure 3). In patients treated with PHMG-P, *T. denticola* concentration dynamics showed an irregular pattern, rising by the 2-week time-point, then lowering by 1 month, followed by rapid growth exceeding the initial level by 4 months. In patients from the control group the concentration of *T. denticola* after initial 2-week spurt, reversed to fall by 1 month. The changes within the groups were not significant (*p* > 0.05).

In all three groups the ratio of *T. forsythia* (detected in 94.2% of the samples) decreased within a month and increased thereafter. However, the initial level was not regained (Figure 3). The changes were considered significant in the CHX group (*p* ≤ 0.05).

*F. nucleatum* was found in every patient engaged in the study. Mechanical debridement and irrigation with CHX or PHMG-P inhibited microbial concentration during the first month, but the initial level was nearly recovered by 4 months (Figure 3). Mechanical treatment coupled to flushing of periodontal pockets with water reduced the bacterial titer at the 2-week follow-up, but further treatment had a limited effect and the concentration of *F. nucleatum* came back to approximately the original level.

*A. actinomycetemcomitans* presence were detected in 55.3% of total samples. Bacterial level increased during the first fortnight. The *A. actinomycetemcomitans* count increased even further in the water group; however, it decreased in the groups treated with antiseptics (Figure 3). Later *A. actinomycetemcomitans* count decreased to below the baseline level in all three groups. The changes within treatment groups were not significant (*p* > 0.05).

*P. intermedia* was detected on average in 59.6% of the samples. Initially *P. intermedia* concentration varied from patient to patient, but the differences were not statistically significant (*p* > 0.05) (Figure 3). During the study *P. intermedia* counts decreased in all treatment groups, albeit not critically (*p* > 0.05). In the control group, mechanical debridement combined with irrigation of the pockets with water diminished the levels of microorganisms by the 2-week check-up, but they successfully recovered by 1 month and further treatment ultimately reduced *P. intermedia* population close to the figures typical for other groups.

## 4. Discussion

The study is based on a double-blind clinical trial assessing the effect of adjunctive irrigation of periodontal pockets with antiseptics during repeated sessions of mechanical debridement. The current longitudinal study provides an insight into the effect of several sessions of non-surgical periodontal treatment on the profile of GCF biomarkers and the bacterial burden in periodontal pockets in relation to clinical status. To estimate the short-term effects, GCF and bacterial samples were collected every fortnight: at baseline, 2 weeks and 1 month after startup; and were taken by 4 months to evaluate mid-term efficiency. Further samples were not analyzed since we did not expect any differences with the available data.

A limitation to the study might be the choice of only selected periopathogens rather than total microbiota as investigation target fundamental for understanding etiology and pathogenesis of the disease. However, testing in vivo anti-microbial efficiency of the antiseptics could be performed relying on few microorganisms.

The trends in total protein production did not agree with biomarker concentrations. Thus, the changes in cytokine levels may be regarded as specific.

Although concentration of pro-inflammatory cytokines in CHX group ceased to rise by 2 weeks, increase in MMP-8 levels observed in CHX group was similar to the dynamics in the PHMG-P and water groups. The ascent of MMP-8 concentration 2 weeks after baseline treatment may be associated with mechanical injury of the tissues due to SRP and release of bacterial LPS into the periodontium. Later decrease in MMP-8 level coincided in time with the reduction of plaque index (PI) and ratios of *P. gingivalis*, *T. forsythia*, and *P. intermedia* cultures [22]. In contrast to the 2-week examination, MMP-8 concentration did not increase following the second treatment session at the 1-month time point. This may be explained by the treatment effect, improved oral hygiene, decreased number of red and orange complex microorganisms and LPS burden. Additionally, CHX could inhibit the activity and auto activation of MMP-8 [36].

The dynamics of IL-1β and MMP-8 levels were resembling in the PHMG-P and water groups, supporting previous findings that MMP-8 is driven by the cytokines [8]. Our study in vitro assessing the effects of CHX and PHMG-P on the secretion of inflammatory mediators by human gingival fibroblasts demonstrated that CHX was able to arrest accumulation of pro-inflammatory cytokines IL-6, -8, and MMP-1 [15,37]. In the present study CHX action, in contrast to PHMG-P treatment, did not induce an upsurge of IL-1β, IL-8, and IL-17A levels, implying that the cytokines role is not the only regulating mechanism of MMP-8 synthesis.

It was earlier reported that biomarker levels can exhibit considerable variability. In the present study, diverse concentrations of cytokines were recorded, resulting in high SD values and an variegated response to therapy [38]. Lack of significant effect of initial periodontal treatment on a wide range of cytokines, was demonstrated apart from granulocyte-macrophage colony-stimulating factor [39]. In the course of periodontal pathogenesis cytokines function in a complex network of overlapping intermolecular interactions, hence the progress of pathological process of periodontal disease is likely to be far more intricate than previously assumed [40].

Lower in *P. gingivalis*, *T. forsythia*, and *P. intermedia* counts coincided with improved clinical parameters, like PI, bleeding on probing (BOP), and pocket probing depth (PPD) [22].

The reduction of *P. gingivalis* counts occurred over 2 weeks, although it was not accompanied by suppression of cytokines in the PHMG-P and water groups. The persistence of elevated cytokine levels may be due to mechanical trauma during debridement and the effect of LPS present in dental plaque, enhancing the original inflammatory response.

Changes in the *P. gingivalis* and *T. forsythia* counts followed the similar patterns in all treatment groups. Growth inhibition taking place up to 1 month caused by debridement every fortnight, was succeeded by active regrowth afterwards. In the absence of intervention, the bacteria re-populated the biotope.

The finding that A. *actinomycetemcomitans* species was not fully compatible with the other bacterial cultures might be explained by the fact that *A. actinomycetemcomitans* grew and occupied vacant niches but was further displaced as the share of periopathogens in oral microbiota was restored.

It was found that *F. nucleatum* recovered to a greater extent than other bacteria. Moreover, *F. nucleatum,* as an essential microbe, promoted binding of anaerobes to the plaque and facilitated biofilm growth and maturation [41]. The three-month period between treatment sessions was long enough for bacteria to re-inhabit the pockets.

The microbial titer of every studied periopathogen in subgingival biofilm varied considerably between patients. This phenomenon of inter-subject heterogeneity in subgingival microbial profiles has been recently reported [42]. The treatment caused a similar inhibiting effect on the tested bacteria, so that no statistically significant differences between treatment groups were revealed (*p* > 0.05). At the same time within one of the antiseptic groups, either PHMG-P or CHX, there were significant changes in *P. gingivalis* and *T. forsythia* counts (*p* ≤ 0.05).

All studied microorganisms presumably played different roles in the development of inflammation. *P. gingivalis* and *T. forsythia* evidently were assigned key parts because all patients were heavily contaminated with these pathogens. CHX and PHMG-P acted in the similar manner against these microorganisms (*p* > 0.05). *F. nucleatum* behaved like a ‘typical’ microbe: its concentration fell during the treatment session but returned to about the original level afterwards.

During the clinical study, irrigation with polyhexamethylene guanidine phosphate significantly reduced pocket probing depths in 4–6 months, but with no significant effect on the mean pocket depth during a year, while no adverse effects of the treatment were observed [22].

Post therapeutic decline in LPS concentration coincided in time with the arrest of inflammation, decrease of pro-inflammatory cytokines, and eventual improvement of clinical parameters, namely reduced PPD, BOP, and PI [22]. The plausible mechanism of action of observed changes could be explained by anti-microbial properties of the antiseptics, mechanical flushing out of the pockets by the liquid flow and mechanical debridement.

## 5. Conclusions

High variability was observed in the levels of inflammatory biomarkers and bacterial cell titers. Adjunctive antiseptic treatment resulted in changes of biomarkers and bacterial counts in the course of the study. The limited effect of adjunctive antiseptic irrigation of the applied protocol were stated.

## Figures and Tables

**Table 1:** Oligonucleotide sequences used in related research and our modifications

Species	Sequences	Our Modifications
*P. gingivalis* [28]	Forward TGGTTTCATGCAGCTTCTTT	-
	Reverse TCGGCACCTTCGTAATTCTT	-
	Probe FAM-CGTACCTCATATCCCGAGGGGCTG-BHQ1	-
*T. denticola* [28]	Forward CCTTGAACAAAAACCGGAAA	-
	Reverse GGGAAAAGCAGGAAGCATAA	-
	Probe FAM-GAGCTCTGAATAATTTTGATGCA-BHQ1	-
*F. nucleatum* [26]	Forward GGATTTATTGGGCGTAAAGC	GG[LNA-A]TTTATTGGGCGTAAAGC
	Reverse GGCATTCCTACAAATATCTACGAA	-
	Probe FAM-CTCTACACTTGTAGTTCCG	CTCTACACTTGTAGTT[LNA-C][LNA-C]G
*T. forsythia* [29]	Forward GAGGTTGTGGAAGGTATG	-
	Reverse GTAGATCAGAATGTACGGATT	-
	Probe FAM-TCTCCGCTTATTTCGTGAC-BHQ1	-
*A. actinomycetemcomitans* [29]	Forward GCGAAACGAAGAGAAGCAAG	-
	Reverse CCTACCCAACAGGCGTATCA	-
	Probe FAM-ATTCCCAACCGCACTT-BHQ1	FAM-ATTCCCAAC[LNA-C]GCACTT-BHQ1
*P. intermedia* [27]	Forward TGTCGGTTTACTGGCTATGTTCTC	-
	Reverse CTTGTCTGTTGGCCATCTTGAAG	-
	Probe FAM-TCAAAGACGCACGTACCAATCCAGACC-BHQ1	-

**Table 2:** Comparison of reaction efficiency and R^2^ values in the present study vs. previous literature reports

Target Microorganism	Eff. (Current)	Eff. (Literature)	R^2^ (Current)	R^2^ (Literature)
*P. gingivalis*	96.5%	91.0% [28]	0.997	0.999 [28]
*T. denticola*	99.3%	97.0% [28]	0.998	1.000 [28]
*F. nucleatum*	99.7%	90–100% [31]	0.996	0.997 [31]
*T. forsythia*	97.0%	88.0% [29]	0.998	1.000 [29]
*A. actinomycetemcomitans*	93.5%	73.7% [29]	0.999	0.999 [29]
*P. intermedia*	95.6%	121.2% [27]	0.994	0.995 [27]
